# Suspension—American Dental Weekly

**Published:** 1898-11

**Authors:** 


### Suspension—American Dental Weekly.

We very much regret to learn that the *American Dental Weekly* has been, for the time being at least, suspended after one year's work.

The *Weekly* was a bright, lively paper. We realized, from the beginning, that it was a very heavy task, and would be found such by the editor and publisher as he progressed with the work. We trust a way may soon be opened for its resumption.

There is room for such a journal, and it ought to be sustained by the profession. It would require for its success, however, two or three editors, in addition to the publisher. We realized at the outstart that Bro. Catching had more upon his hands than any one man could do, and especially one so particular as Bro. Catching.

